# Supplementary material for: Are carbon emissions trading and green financial instruments synergistic? -Comprehensive quantitative research based on content analysis
Source: PLoS One. 2024 Mar 7;19(3):e0298601. doi: 10.1371/journal.pone.0298601 (PMC10919601; doi:10.1371/journal.pone.0298601)
Supplement: S1 Data — (ZIP) [file pone.0298601.s001.zip › Data/地级市Z_F工作报告.zip!/░▓╗╒/┬φ░░╔╜╩╨/└┤╫╘╣1⁄2╓┌║┼í╛┬φ┐╦╩2╛▌═°í┐_2018.docx]

# **政府工作报告（2018）**

【字体大小：[大](http://zwgk.mas.gov.cn/openness/detail/content/javascript:doZoom(18)) [中](http://zwgk.mas.gov.cn/openness/detail/content/javascript:doZoom(15)) [小](http://zwgk.mas.gov.cn/openness/detail/content/javascript:doZoom(14))】

——2018年1月6日在马鞍山市第十六届人民代表大会第一次会议上

市长  左俊

各位代表：

    现在，我代表市人民政府，向大会报告政府工作，请予审议，并请市政协委员和其他列席人员提出意见。

    一、过去五年的工作

    市十五届人大一次会议以来的五年，是马鞍山发展进程中很不平凡的五年。五年来，我们在省委、省政府和市委的坚强领导下，坚持以习近平新时代中国特色社会主义思想特色社会主义思想为指导，全面贯彻落实党的十八大、十九大精神和习近平总书记视察安徽重要讲话精神，坚持稳中求进工作总基调，克难攻坚、锐意进取，经济社会发展取得了显著成效。

    ——综合经济实力迈上新台阶。预计地区生产总值由2012年的1233.9亿元增至2017年的1720亿元，年均增长9.6%，总量有望进入全省前三。城镇和农村居民人均可支配收入分别由27142元、11838元增至41380元、19310元，年均分别增长8.8%、10.3%，总量均居全省第一。

    ——产业转型升级成效显著。智能装备（机器人）、轨道交通装备、高端数控机床产业基地先后获批为国家级、省级战略性新兴产业基地；新能源、电子信息、生物医药等战新产业快速成长。战新产业产值占规上工业总产值比重由14.5%增至18.2%。服务业增加值年均增长9.2%。三次产业结构由5.9:66.4:27.7调整为5.3:57.4:37.3。

    ——重大基础设施相继建成。马鞍山长江大桥、宁安城际铁路和常合、滁马、巢无高速建成通车，马濮旅游大道、314省道与南京成功贯通；郑蒲港开埠运营；商合杭高铁含山段、郑蒲港铁路加快建设。新三馆、市体育会展中心、高铁马鞍山东站建成运营。有序推进合巢马城际铁路、轨道交通1号线等项目前期工作。

    ——对外开放水平不断提升。马鞍山综合保税区获得国务院批复，并顺利通过省级预验收；口岸扩大开放通过国家验收，肉类指定口岸启动运营，粮食指定口岸获批筹建；安徽江海联运枢纽中心建设迈出坚实步伐。海峡两岸产业转型转移示范基地和节能环保产业合作园获批设立；开辟对欧合作新通道，中欧产业合作园启动建设。

    ——民生工程实事成果丰硕。脱贫攻坚深入推进，29648名贫困人口稳定脱贫、28个贫困村成功出列；56项民生工程、30件民生实事全面完成，267个棚户区、94个老旧小区完成改造；5016名困难职工脱困解困；农村环境“三大革命”在全省率先实施；主要河湖水质良好，PM10平均浓度下降39%，马鞍山天更蓝、水更绿、空气更清新。

    ——城市综合品质持续改善。实现全国文明城市“四连冠”、全国科技进步先进市“八连冠”、全国双拥模范城市“三连冠”、国家卫生城市再度蝉联。荣获全国健康城市试点市、全国国土资源节约集约模范市、国家公共文化服务标准化示范市、全国和谐社区建设示范城市等十多项国字号荣誉。国家创新型城市、国家森林城市、全国质量强市示范城市创建加快推进。

    五年来，我们主要做了以下工作：

****（一）全力稳增长促转型。****促进投资和项目建设。累计完成固定资产投资9261亿元，年均增长13.2%。大力招商引资，累计实际利用外商直接投资95.6亿美元、省外亿元以上项目到位资金3296亿元。促进工业经济转型升级。新增规上工业企业827户、总量达1228户，规上工业增加值年均增长10.8%。钢铁、化工、食品等传统产业加快技术改造，累计实施技改项目983个、完成投资1555.7亿元。马钢成为全市转型升级排头兵，多元化发展取得重要进展。促进服务业提速发展。实现社会消费品零售总额2060亿元、年均增长15%，万达、金鹰等商贸综合体建成运营。文旅产业融合发展，采石矶5A级景区加快创建，6家景区晋升4A级，启迪乔波室内滑雪场等40个项目建成运营，旅游业总收入年均增长20.1%。促进农业提质增效。粮食总产稳步提升，农产品加工产值达到470亿元，年均增长13.7%，土地流转率达到60.2%；家庭农场和农民专业合作社分别达到407个、265个，新增“三品一标”产品130个，和县获批创建国家现代农业产业园。促进区域经济协调发展。市经开区、慈湖高新区在全国位列81位、56位，郑蒲港新区港产城一体化加快推进，示范园区加快建设。当涂县位列全国百强县72位、提升4个位次，含山县、和县经济总量占全市比重提升2.5个百分点。花山区、雨山区、博望区加快发展，占全市经济比重达37.1%。

****（二）深入推进改革开放。****全面落实“三去一降一补”。2016年以来马钢压减钢铁产能265万吨，“三供一业”分离移交有序推进。2017年末商品房销售面积、金额同比增长32.6%、37.1%，去化周期10.1个月，两年减少17.2个月，土地出让金收入105.7亿元、同比增长73%。上市挂牌企业从9家增加到93家，直接融资年均增长67.47%。降低企业制度性交易成本和税费负担，规上工业企业每百元主营业务收入成本降低3.3元。“放管服”、商事制度改革不断深化，“双随机、一公开”监管全覆盖，“多证合一”全面实施，“最多跑一次”“不见面审批”“窗口无否决权”“微笑服务”创新推进，营商环境进一步优化。农村集体产权制度改革稳步推进，国有林场改革任务全面完成。

    深入实施创新驱动发展战略。全社会研发经费投入占GDP比重达2.61%，万人发明专利拥有量由2.32件增加到16件，创新能力居全省前列。累计培育认定科技“小巨人”企业200家，高新技术企业达348家、净增191家。人才集聚“313行动计划”有效推进，引进各类人才13780人、高层次人才团队88个。新增工程技术研究中心等市级以上研发平台240个，组建各类众创空间24家。大力实施开放带动战略。坚持东向发展，成功加入长三角城市群。定期举办海峡两岸电子信息博览会、节能环保产业论坛，台商服务中心建成启用；全市台资企业216户、欧资企业40户。马钢、十七冶等骨干企业积极参与“一带一路”建设，开展国际产能合作。全市累计实现外贸进出口总额162.2亿美元、外经营业额19.2亿美元。

****（三）持续提升城市品质。****不断完善城市功能。城市总体规划修编获国务院批准，中心城区控制性详规全覆盖。成为全国城市设计试点市。城市路网管网不断完善，新增城市道路超60公里，污水管网改造364公里，综合管廊开工建设；改造提升国省干线8条、212.7公里。海绵城市试点稳步推进。进一步加强城市治理，“三线三边”“两治三改”“四整治四提升”等专项行动压茬实施，当涂县首获全国县级文明城市。智慧城市建设加快推进，数字化城管建成运营，智慧交通综合信息中心、公交电子站牌全部建成，智慧小区建设率先启动。持续建设美丽乡村。16个乡镇政府驻地、96个省级中心村、40个市级中心村全面建成，当涂县、和县被评为全省美丽乡村建设先进县。农村畅通工程加快推进，改造县乡公路1836公里，新建、改造农村桥梁116座。改造农村危房20064户。

****（四）狠抓生态环境保护。****加强资源节约集约利用。节能减排任务全面完成，单位生产总值能耗下降24.9%，主要污染物排放总量控制在省下达指标内。土地利用集约高效，新增工业用地亩均投资超200万元。推进资源循环利用，慈湖高新区获批国家资源综合利用“双百工程”示范基地，生活垃圾焚烧发电、光大生物质能发电项目建成投产。推进生态强市建设。创建省级以上生态县区3个、生态乡镇29个、生态村63个、绿色社区16个。河长制、林长制等长效机制全面推行。制定长江经济带生态优先绿色发展实施意见，共抓大保护、不搞大开发；千万亩森林增长工程全面完成，接续实施林业增绿增效增美行动。

    加大生态环境保护执法力度。全面启动大气、水、土壤污染防治行动计划，长江沿岸化工、造纸等企业实时监控、达标排放；农业面源污染治理得到加强，农药、化肥使用量分别下降5.7%和12.5%。中心城区水环境综合治理加快推进，25条黑臭水体完成整治。

****（五）聚力聚焦民生保障。****就业保险持续加力。城镇新增就业26万人，城镇登记失业率控制在3%以下。“全民参保登记计划”全国试点顺利开展，新型农村合作医疗、大病保险实现全覆盖，城乡居民养老、医疗保险应保尽保，红十字和慈善公益事业稳步发展。教育卫生事业快速发展。教育综合改革不断深化，学前教育三年行动计划顺利完成，普惠制幼儿园比例超85%；市二中郑蒲港分校、文天学院郑蒲港校区、安工大工商学院当涂校区建成招生。卫生计生服务均等化水平不断提升，“全面两孩”政策平稳实施；与丰盛集团、三胞集团深入合作，智慧养老服务体系加快构建。文化体育事业蓬勃发展。基本建成市县乡村四级公共文化服务体系；连续举办中国李白诗歌节、成功举办第四届中国文化馆年会。新闻出版、广播电视、社会科学、档案和地方志工作水平不断提升；全民健身运动大力开展，我市运动员孟苏平在里约奥运会上勇夺金牌。气象、地震、防灾减灾工作取得新进展。工会、共青团、妇女儿童事业扎实推进。

****（六）提升社会治理能力。****村居“大管家”全覆盖，创建“精品”“四优”社区64个。“平安马鞍山”建设不断深入，安全生产“铸安”行动持续开展，安全生产形势保持稳定，含山县荣获全国平安建设先进县；矛盾纠纷多元化解“马鞍山经验”在全国推广，信访秩序持续好转，社会治安防控体系不断完善。众志成城战胜了60年一遇洪涝灾害。民族和宗教工作得到加强。人民武装、国防动员、拥军优属和军民融合工作深入推进。

    五年来，我们立足于更好发挥政府作用，政府自身建设取得新进展。加强政治能力建设，牢固树立“四个意识”， 群众路线、“三严三实”“两学一做”“讲看齐见行动”“讲重作”等专题教育活动扎实开展。加强党风廉政建设，全面落实管党治党主体责任和“一岗双责”，严肃党内政治生活，加强权力监督和风险防控，加大正风肃纪力度，政府系统问责处理661人。加强政府作风建设，严格执行中央八项规定和省、市有关规定，严格控制机构编制、预算追加、一般性出国访问以及政府债务。加强政府法治建设，建立政府学法、法律顾问和公职律师制度，重大事项民主公开科学决策；主动接受人大依法监督、政协民主监督，高质量办理人大议案建议616件、政协提案1381件；充分发挥民主党派、工商联、无党派人士以及人民团体参政议政作用。

    刚刚过去的2017年，是本届政府的收官之年。经过全市上下共同努力，较好完成了市十五届人大七次会议确定的主要目标任务。一年来，我们着力强化经济运行调度，围绕稳增长、调结构、补短板、强弱项，及时制定政策措施，保持经济平稳较快增长，前三季度在全省综合考核中位居第二。我们着力提高经济发展质量和效益，财政收入增长10.1%，税收收入增长20%、占财政收入比重提高7个百分点；金融存贷款分别增长8.5%、12.6%。我们着力促进实体经济发展，深入实施“四送一服”双千工程，马钢、华菱星马、山鹰纸业、蒙牛、达利、金星钛白以及十七冶、中冶华天等重点骨干企业增势强劲、产销两旺，规上工业企业利润增长1.2倍。我们着力开展“项目建设年”活动，大力招商引资，成功引进摩利纳轨道交通、奥克斯空调、沈阳机床、泰能新能源、创久集成电路、欢颜机器人、豪森药业、顺丰产业园等一批重大项目。我们着力推进创新发展，深化与中国工程物理研究院、清华大学、南京大学等十余家大院大所战略合作，顺利完成“8533”科技创新目标任务。我们着力深化改革，出台改革方案87项，14项形成初步成果，其中城市管理综合执法体制改革、公立医院改革、国资国企改革等在全省领先。我们着力推动开放联动发展，出台等高对接苏浙沪全面融入长三角、对台对欧经贸合作意见，推进对接南京都市圈、合肥都市圈“双十”工程。我们着力打好环保攻坚战，整改问题777项，拆除长江沿岸非法码头82家，关停船舶修造企业22家，关闭搬迁禁养区养殖场户497家，关闭整顿非煤矿山52座，关停取缔化工企业3家，淘汰小散乱污企业326家，全面完成石臼湖围网拆除。我们着力抓稳定、护安全，全年没有发生一起较大以上安全生产事故。

    各位代表！

    过去五年，面对经济下行以及资源型城市转型双重压力，能够取得上述重大改革发展成就，根本在于习近平新时代中国特色社会主义思想特色社会主义思想的科学指引，在于新发展理念在马鞍山的生动实践。回顾五年来的发展历程，我们深切体会到：牢记宗旨、人民至上是发展的根本点；转型升级、加快发展是发展的主旋律；创新驱动、开放联动是发展的主战略；招商引资、招才引智是发展的主抓手；生态优先、绿色发展是发展的生命线；争先进位、开拓进取是发展的精气神。这些宝贵的经验，必须长期坚持、大力弘扬。

    五年来取得的改革发展成就，是省委、省政府和市委坚强领导的结果；是市人大、市政协大力支持依法监督的结果；是历届领导班子一任接着一任干的结果；是全市人民齐心协力、共同拼搏的结果。在此，我代表市人民政府，向全市人民，向市人大代表、市政协委员、离退休老同志，向市各民主党派、工商联、无党派人士、人民团体和各界人士，向中央和省驻马单位，向驻马解放军指战员、武警官兵和政法公安干警，向所有关心、支持、参与马鞍山建设发展的境内外投资者、海内外朋友，表示衷心的感谢和崇高的敬意！

    同时，必须清醒看到，当前我市发展中还存在一些深层次矛盾和问题，政府工作中还存在许多不足和弱项：战略性新兴产业集聚度不高，产业链不完整；城市功能有待进一步提升，重点项目建设需要提速提效；环境整治需要持续用力，生态修复任务繁重；财政平衡压力较大，政府债务负担较重；少数部门“四风”问题不同程度存在，部分领域腐败问题时有发生。对这些问题，我们将采取有效措施，切实加以解决。

    还需要向各位代表报告的是，去年经济形势虽然较好，但规模以上工业增加值、固定资产投资增速未能达到预期计划。这里既有我们积极淘汰落后产能、有序消化历史遗留的问题，更有发展不够充分的问题。今年，我们将在这些方面狠下功夫，努力补上短板。

    二、今后五年政府工作的目标任务

    未来五年是决胜全面建成小康社会、实现第一个百年目标的关键期；也是我市转型升级、加快发展的攻坚期。我们面临着完成“十三五”规划目标，全面建成小康社会走在全省前列，全面开启基本现代化建设新征程等重大历史任务。新时代、新征程、新目标，在前进的道路上，肯定会遇到许多风险和挑战，需要我们付出更为艰苦的努力。同时，也必须看到，当前马鞍山发展的基础、势头、条件、氛围都更好，我们发展的前景是光明的。从外部环境看，世界经济企稳向好，有望跨越新平庸、开启新增长；党的十九大对坚持和发展中国特色社会主义作出战略部署，省委、省政府对建设现代化五大发展美好安徽作出系统安排，全国全省宏观经济将保持良好发展势头。从自身条件看，经过多年努力，我市已经站上了新的发展起点，人均GDP超过1万美元，经济发展已从高速增长转向高质量发展阶段，经济发展的动能正在加速转换。更为重要的是，新时代全市人民对美好生活的向往，必将凝聚起团结奋进的磅礴力量。我们要进一步认清形势、顺应大势，立足新起点、抢抓新机遇，推动新时代马鞍山现代化建设不断取得新胜利。

    今后五年政府工作的指导思想是：全面贯彻落实党的十九大精神，以习近平新时代中国特色社会主义思想特色社会主义思想为指导，认真贯彻落实省委十届六次全会、市委九届六次全会精神，坚持稳中求进工作总基调，坚持新发展理念，紧扣社会主要矛盾变化，按照高质量发展的要求，统筹推进“五位一体”总体布局和协调推进“四个全面”战略布局，坚持以供给侧结构性改革为主线，坚持以五大发展行动计划为总抓手，统筹推进稳增长、促改革、调结构、惠民生、防风险各项工作，大力推进改革开放，大力推进创新驱动，推动质量变革、效率变革、动力变革，加快建设现代化经济体系，决胜全面建成小康社会，确保打赢防范化解重大风险、精准脱贫、污染防治攻坚战，全力推动“三个走在前列”，建设经济强市、文化强市、生态强市、科教强市和人才强市，全面开创新时代马鞍山现代化建设新局面。

    今后五年发展的主要目标是：经济保持中高速增长，地区生产总值年均增长8.5%以上，经济发展质量效益全省领先，顺利完成“十三五”规划和市第九次党代会确定的目标任务。到2022年，在全面建成小康社会的基础上，继续前进，基本建成社会主义现代化迈出坚实步伐，总量居全省前三，人均主要指标居全省前列，努力达到或接近长三角城市群平均水平。

    实现上述目标，今后五年要聚力实施“五大发展行动计划”：

****（一）聚力实施创新发展行动计划。****将创新作为第一动力，打造国家创新型城市。统筹推进“三重一创”，培育壮大新动能，集聚发展智能装备（机器人）、轨道交通装备、高端数控机床三大战新产业基地，大力发展新能源、电子信息、生物医药等市级战新产业。突出高端、智能、绿色、精品、服务型制造，大规模改造提升传统产业。推进钢城融合和马钢多元化发展。加快发展现代服务业，着力推动产业结构迈向价值链中高端，力争高新技术产业增加值占规上工业增加值比重达50%，第三产业增加值占GDP比重超过40%。实施人才强市战略，汇聚高端人才，造就企业人才，培养技能人才，促进各领域人才竞相涌现。与更多的大院大所加强战略合作，完善以企业为主体、市场为导向、产学研深度融合的技术创新体系，全社会研发经费投入占GDP比重达3%以上。全要素劳动生产率明显提高，加快构建以创新为战略支撑的现代化经济体系。

****（二）聚力实施协调发展行动计划。****将协调作为内生特点，推进城乡协调、区域联动，打造全省均衡发展先行区。坚持“组团式、网格化、田园城市”建设思路，推动主城区东扩南进跨江发展，促进秀山新区加快发展，加快老城区改造、老工业区搬迁和独立工矿区综合治理，推进郑蒲港新区港产城一体化发展，提升县城、小城镇承载力，建成智慧城市。发挥县区、园区经济建设主战场作用，推动含山、和县、博望加快发展，支持当涂、花山、雨山优化发展；推动开发区改革创新发展，坚持做大体量、做强特色、做优结构，鼓励市经开区、慈湖高新区在全国争先进位，支持当涂经开区迈入国家级，努力打造1-2个千亿级园区。坚持农业农村优先发展，实施乡村振兴战略，培养造就一支懂农业、爱农村、爱农民的“三农”工作队伍，进一步推动农业强、农村美、农民富。

****（三）聚力实施绿色发展行动计划。****将绿色作为普遍形态，扎实推进生态强市建设，打造生态文明建设马鞍山样板。推进经济绿色转型，健全绿色低碳循环发展的经济体系，形成节约资源和保护环境的空间格局、产业结构、生产方式、生活方式，能源资源利用率稳步提高，主要污染物排放总量持续下降。坚决打好蓝天保卫战、碧水攻坚战、净土持久战，深入实施城乡人居环境整治行动，建成国家森林城市，显著改善全市生态环境质量。加强生态文明制度建设，完善生态文明建设法规规章，健全生态补偿与市场化机制，深化生态环境监管体制改革，坚决制止和惩处破坏生态环境行为。

****（四）聚力实施开放发展行动计划。****将开放作为必由之路，推动开放联动走在全省前列，打造内陆开放新高地。全面建成“一区一港多口岸”开放平台，积极参与创建中国（安徽）自由贸易试验区，主动跟进融入自由贸易港建设。高水平建设海峡两岸产业转型转移示范基地和节能环保产业合作园，建成中欧产业合作园。深化与长三角区域合作，深度融入南京都市圈、合肥都市圈，推动重大基础设施互联互通，建成郑蒲港铁路，开工建设合巢马城际铁路，深化轨道交通线网规划和建设规划。坚持走出去和引进来相结合，培育对外贸易新优势，拓展对外投资新领域，创新招商引资新模式，打造更高质量外向型产业新体系。

****（五）聚力实施共享发展行动计划。****将共享作为根本目的，不断满足人民群众日益增长的美好生活需要，打造宜居宜业幸福城市。优先发展教育事业，全面建成健康城市，居民收入与经济增长保持同步，统筹推进就业、养老、住房以及社会救助等各项社会事业，在幼有所育、学有所教、劳有所得、病有所医、老有所养、住有所居、弱有所扶上取得更大进展。坚决打赢脱贫攻坚战，确保到2020年，现行标准下农村贫困人口全部稳定脱贫。公共服务体系更加健全，社会文明程度显著提高。大力推进文化强市建设，繁荣发展文化事业，实施文化惠民工程，将文旅产业打造成全市支柱产业。深化“平安马鞍山”建设，深入推进依法治市，维护社会和谐稳定。

    三、2018年主要工作

    今年是贯彻落实党的十九大精神的开局之年，是改革开放40周年，也是新一届政府的开局之年，做好今年各项工作意义十分重大。全市经济社会发展主要预期目标为：地区生产总值增长8.5%以上，财政收入同步增长，固定资产投资增长8%左右，社会消费品零售总额增长10%，城镇居民人均可支配收入增长8%，农村居民人均可支配收入增长8.5%，城镇登记失业率控制在4%以下，单位GDP能耗、主要污染物排放总量控制在省下达指标以内，有力有效防控金融债务风险。这一预期目标的设定，体现了质量第一、效益优先的鲜明导向，同时也兼顾了“十三五”规划的有序实施。

    我们将重点抓好以下十个方面工作：

****（一）深化供给侧结构性改革，大力推进质量变革。****在努力保持经济中高速增长的同时，大力支持实体经济发展，提高供给体系质量。

    优化存量资源配置。坚持用市场化手段化解过剩产能，完成钢铁行业去生铁产能100万吨、粗钢产能128万吨。引导企业兼并重组20户。加快传统产业优化升级，实施亿元以上技改项目100个，完成投资200亿元。继续开展“四送一服”双千工程，进一步降低企业成本，力争工业项目供地8000亩；促进金融更好服务实体经济，新增上市挂牌企业30家，完成直接融资220亿元、社会融资408亿元。

    扩大优质增量供给。深入开展“项目建设年”活动，推进760个重点项目建设，完成投资950亿元，其中市级调度的100个重点项目完成投资300亿元。推动泰尔智能产业园等224个项目开工建设，埃斯科特钢等135个项目竣工投产，特别是摩利纳轨道交通、奥克斯空调、沈阳机床、泰能新能源、永途汽车等重大高端制造业项目，是转型升级的排头兵、加快发展的增长点、区域发展的竞争力，必须全力精准服务，推动快开工、快建设、快投产。激发民营经济发展活力，放宽市场准入、优化营商环境，力争民间投资增长9%。坚持去库存与稳市场并重，加快建立多主体供应、多渠道保障、租购并举的住房制度，保持房地产市场健康发展。政府性投资要坚持量力而行、尽力而为，统筹项目建设时序。狠抓项目调度，及时解决项目实施过程中的资金、征迁、用地等难题。

    加大招商引资力度。强化招商引资“一号工程”理念，坚持全市招商政策一盘棋，集中精力财力，在招大引强上再聚焦、再发力、再突破。创新招商引资方式，围绕重点产业、重点区域和重点项目，推进基金招商、产业链招商、第三方招商。开辟“绿色通道”，全方位服务好项目和企业，促进招商项目早签约、早落地。力争实际利用外商直接投资增长7%，省外亿元以上项目实际到位资金增长8%。

    完善城市基础设施。构建“一江两岸”快速交通体系，力争S367马鞍山段、G347和县段改建工程尽快完工，加快S442博望段、S210和城段改建工程建设，积极推进S446澄湖路等前期工作，完成农村公路畅通工程310公里。加快推进长江马鞍山段二期整治、裕溪河综合治理等重大水利工程。大力推进秀山医院、综合客运枢纽中心、市妇幼保健院计划生育服务综合楼加快建设，市残疾人康复中心投入使用。完善城市内部路网，打通断头路，实施湖西路等道路改造提升工程，加快银杏大道东延，开工江东大道五期。完善供水、燃气、通信、电力、消防等管网设施；实施宁芜铁路道口“平改立”工程，建设公共停车场6个。

****（二）推进创新型城市建设，加快推进动力变革。****把创新摆在发展重要位置，坚持以创新引领经济发展，加快新旧动能转换。

    大力支持企业自主创新。继续实施科技创新券、所得税优惠、研发费用加计扣除、后补助等激励政策，引导企业加大研发投入、培养研发人员、购置研发设备、建设研发平台。加大创新型企业培育力度，新培育科技“小巨人”企业80家，新认定高新技术企业50家，高新技术产业增加值增速保持在15%以上。全市研发经费投入占GDP比重达2.65%。

    加快创新平台建设。深化与知名企业、大院大所、高等院校合作，新组建研发机构20家以上；强化招才引智政策举措，吸引大学毕业生来马就业创业，引进培养高端领军人才和高技能人才6000人，培育招引高层次科技人才团队20个。健全科技创新平台体系，重点推进1家以上院士工作站、3家产业技术创新战略联盟等平台建设。推动政产学研深度融合，加快科技成果转移转化，完成技术合同交易额12亿元以上。推动军民融合发展，促进军转民、民参军。

    推进创新要素集聚。优化全市创新资源配置，引导创新创业平台差异化发展。坚持产业链、创新链、价值链、资金链“四链合一”，加快建设众创空间、孵化器、加速器，努力把成长型、种子型企业引进来、培育好。推动科技金融融合，继续开展专利权质押贷款和科技保险工作，实施好首担、首投、首贷、首补。继续办好全球创客（马鞍山）大赛。创建国家知识产权示范市，强化知识产权创造、保护、运用。

****（三）加快产业转型升级，着力建设现代化产业体系。****坚持转型发展，推动产业优化升级，努力实现产业层次中高端。

    发展先进制造业。继续实施制造业升级行动计划，新增规上工业企业110家，规上工业增加值增长9%。实施战略性新兴产业集聚发展工程，坚持基地建设项目化、项目工作责任化，狠抓基地重点项目建设，实现战新产业产值增长18%。继续实施质量品牌升级工程，成功创建全国质量强市示范城市。

    发展现代服务业。研究出台支持服务业集聚区发展政策，加快14个现代服务业集聚区建设，服务业增加值增长9%以上。积极开发适应消费升级新产品，重点培育健康养老、教育培训等新型消费热点；大力发展互联网、物联网、大数据、云计算、现代物流、工业设计、人力资源开发等生产性服务业。推进商贸业提档升级，新增限上商贸企业30家。物流标准化建设全面完成，开工建设顺丰产业园；做大做强马鞍山软件园、青年电子商务产业园。

    发展现代农业。实施农业产业化行动计划，农产品加工产值增长8%。高质量建设国家级、省级现代农业示范园区。加强“三品一标”农产品认证，扩大含山大米、和县蔬菜、当涂水产等特色农业品牌影响力。积极发展乡村旅游，推进农村一二三产融合发展。拓展现代农业经营体系，新增新型农业经营主体50个。

****（四）加快开放联动发展，构建开放型经济体系。****充分发挥马鞍山区位优势，坚持东向发展，扩大对外开放。

    加强对外合作。深化对台合作，密切人员往来，巩固与台湾知名工商协会经贸合作关系，高水平建好重大招商平台；服务好茂迪、正崴等一批台资企业，力争对台合作再上新台阶。深化认识、营造氛围，构建管道、建立平台，扩大对欧合作成果。支持企业参与“一带一路”建设，鼓励有条件的企业在境外设立生产基地、开展海外并购，加大对中小进出口企业的扶持力度，完成进出口总额33亿美元、外经营业额3.2亿美元。

    全面融入长三角。把握长江经济带发展战略机遇，工程化、项目化推进与南京都市圈、合肥都市圈深度融合。协调推进商合杭高铁含山段加快建设，加快推进合巢马城际铁路、宁马高速公路改扩建、G329郑巢路等工作步伐。推动芜马宣城市组群发展，支持宿马产业园加快建设。

    推进以港兴市。综合保税区正式封关运行；建设粮食指定口岸，申建进境水果、冰鲜水产品等指定口岸，构建“口岸+产业+市场”一体化的口岸经济区。加强港口基础设施和长江沿岸砂石集散中心等集疏运体系建设，完成郑蒲港二期工程申报；深入推进大通关建设，全面开展“单一窗口”工作。持续推进与上海、合肥、舟山、太仓等港口的紧密合作，积极支持配合全省港口管理体制改革。

****（五）实施乡村振兴战略，加快县域经济发展。****把乡村振兴、县域振兴作为解决发展不平衡的关键，研究出台实施乡村振兴战略的具体意见，建立健全城乡融合发展体制机制，提升协调发展水平。

    深化农村综合改革。落实承包地“三权”分置政策，探索土地确权成果利用有效模式，健全县乡村三级土地流转服务网络，大力推进适度规模经营。全面开展农村集体经济组织清产核资，实施“抓股改、促三变”改革，推进35个村改革试点。实施农村金融改革创新，推动农业保险、“劝耕贷”农村全覆盖。推进水利管护体制、供销社和国有粮食企业改革。

    加快美丽乡村建设。推进13个美丽乡镇、77个中心村整治建设。补齐农村基础设施短板，实施农村饮水安全、“四好”农村道路、电网改造、义务教育、电商全覆盖巩固提升五大工程。继续推进全域环境整治、农村环境“三大革命”，农村生活垃圾无害化处理率达90%以上，推行农村垃圾分类和资源利用试点，确保所有乡镇污水处理设施投入运营、发挥效用。加强农村基层基础工作，健全自治、法治、德治相结合的乡村治理体系。

    促进县域经济发展。按照市县一体、城乡一体发展理念，加强规划、基础设施、产业、公共服务的无缝对接，进一步提升全域马鞍山融合发展水平。按照主体功能区规划，探索各具特色县域经济发展之路，加快培育特色产业集群，做强主导产业和首位产业。按照“一县一区”原则，稳妥推进开发园区整合。按照新型城镇化要求，提高人口集聚度。大力支持黄池、香泉、乌江等国家和省级特色小镇建设，加快中心镇建设。

****（六）深化重点领域改革，增强发展内生动力。****积极稳妥深化改革，周密制定改革计划，抓好已出台重大改革方案的落地。

    深化放管服改革。推动企业登记全程电子化和电子营业执照发放工作。开展“减证便民”专项行动，大力支持大众创业、万众创新。深化互联网+政务服务，建成覆盖市县乡村四级政务服务“一张网”，让数据多跑路、群众少跑腿。加强村居“大管家”和基层为民办事场所标准化建设。

    深化行政体制改革。开展县属生产经营类事业单位转企改制。深化城市执法管理体制改革，推动“智慧城管”建设，推进基层城管执法队伍正规化、标准化。稳妥推进生态、文化、审计、统计等管理体制改革。

    深化财税金融改革。加强国有资本管理，规范投资和经营行为。严格政府债务管理，开展“四清四实”专项整治，有效防范和化解债务风险，坚决严控隐形债务。严厉打击违法违规金融活动，重点整治乱办金融、非法集资、乱加杠杆等严重干扰金融市场秩序行为。依法理财治税，提高财政资金使用效益。落实环境保护税等税制改革任务。

    深化医药卫生体制改革。按照“政事分开、管办分离”要求，改革公立医院领导体制；建立现代医院管理制度。推进市级医联体建设，建成3个综合性医联体、3个专科联盟。推进县域医共体建设，开展县级牵头医院托管乡镇卫生院、中心卫生院“管理圈”等体制机制创新试点。理顺医保管理体制，改革医保支付方式。

****（七）打好污染防治攻坚战，推进生态强市建设。****坚持生态优先、绿色发展，巩固生态环保长效机制，积极创造更多优美公共生态产品。

    推进重点领域污染防治。不懈怠、不放松，持续抓好中央和省环保督察反馈问题整改，强化长江经济带“共抓大保护”突出问题整治，PM10平均浓度持续下降，重点河流和集中饮用水源地水质全面达标；加强土壤污染管控和修复，推动城市生活垃圾无害化处理全覆盖。开展城乡黑臭水体治理、集中养殖粪污资源化利用、秸秆综合利用提升专项行动。

    推进生态文明制度建设。完成全市生态保护红线划定工作。推动森林生态网络、森林生态屏障、绿色长廊建设，完成各类造林绿化1.8万亩，建成城市绿道11公里。严格落实河长制、林长制。抓好采石国家级风景名胜区、石臼湖自然保护区依法保护工作。

     推动绿色低碳循环发展。大力发展节能环保、清洁生产、清洁能源产业，创建省级绿色食品生产基地5个，新增省级以上绿色工厂3家；推进冶金行业污染防控与资源综合利用重大新兴产业工程。加强土地节约集约利用，处置批而未供、供而未用及低效用地8000亩；做好第三次全国土地调查工作，启动全市土地利用总体规划修编。优先发展公共交通，积极创建省级优先发展公共交通示范城市。

****（八）推动文化持续繁荣，着力建设文化强市。****挖掘文化资源，坚定文化自信，增强文化自觉，进一步提升文明建设水平。

    拓展文明创建。培育和践行社会主义核心价值观，深入开展理想信念教育和中国梦宣传。实施公民道德建设工程、全民科学素质行动计划，加强未成年人思想道德建设。加强革命遗址保护，强化革命传统和爱国主义教育。巩固提升文明城市、文明村镇、文明单位、文明家庭、文明校园创建成果。实施“美丽马鞍山·文明再升级”专项行动，办好第四届好人节和邻里节，弘扬志愿服务文化。

    发展文化事业。打造公共文化服务体系升级版，构建城市“20分钟公共文化服务圈”和城乡公共文化互联互通网络体系。办好第30届中国李白诗歌节，新建100个村居综合文化服务中心，打造100个公共数字文化社区，实现农家书屋数字化全域覆盖。加强文化遗产保护利用，培育50个市级文化名村、5个省级历史文化名镇。广泛开展全民健身运动，积极承办区域性和全国性重大体育赛事。做好社会科学、文学艺术、地方志工作，加快市档案资源服务中心建设。

    加快旅游业发展。推进采石矶创建5A级旅游景区、褒禅山香泉旅游度假区创建国家级、凌家滩创建国家考古遗址公园，支持含山县创建国家全域旅游示范区。加快“创客+”文化创意产业园、中影影视基地等重点项目建设。

****（九）加快发展社会事业，提高民生保障水平。****牢固树立以人民为中心的发展思想，尽心尽力改善民生，努力让发展成果更多惠及人民群众。

    推进民生工程建设。在继续实施好省民生工程的基础上，办好长江大桥市内通行费减免、社区“儿童之家”达标、高层住宅二次供水改造、电梯应急处置服务平台、城市悦书房建设、心理健康惠民工程6件民生实事。新增城镇就业4.3万人。实施棚户区、危旧房、老旧小区改造项目51个。

    打赢脱贫攻坚战。进一步精准脱贫攻坚基础信息，落实落细各项扶贫政策，高质量推进产业扶贫，加强小额担保贷款、财政扶贫资金管理。突出帮扶“三无”特困群体，完善防范返贫机制，解决好非贫困户、非贫困村实际困难。

    优先发展教育事业。继续实施学前教育三年行动计划，支持社会力量举办幼托机构，扩大普惠制幼儿园数量和比例，解决入园难、入园贵的问题。推进集团化办学，促进义务教育均衡优质发展，扩大优质教育资源覆盖面，着力解决“择校热”“大班额”等突出问题。普及高中阶段教育，支持博望高中建设，实施红星中学搬迁，做好中考改革试点和新高考改革工作。推进“医教结合”国家特殊教育改革试验区建设。大力支持职业教育体系建设，推动产教融合、校企合作。

    提升社会保障水平。深入推进健康马鞍山建设。全面实施全民参保计划，城镇职工社保参保率达98%以上，城乡居民综合参保率达92%以上。健全统一的城乡居民基本医疗保险制度，探索建立长期护理保险制度，完善大病保险政策，提高跨省异地就医直接结算效率。健全最低生活保障、失地农民生活保障标准动态调整机制，完善社会救助体系和城乡居民临时救助制度。加强对妇女、儿童、青少年的权益保护，健全农村留守儿童、老人关爱服务体系；发展红十字、慈善等公益事业。坚持医养结合，推进养老服务体系建设三年行动计划。

****（十）加强社会治理，打造共建共治共享体系。****推动实现政府治理和社会调节、居民自治的良性互动，提升社会治理水平。

    加强城市综合治理。充实大数据平台，整合优化全市热线服务，推动城市管理精细化、智能化。健全社会矛盾纠纷排查预警机制，完善调解、仲裁、行政裁决、行政复议、诉讼等有机衔接、相互协调的多元化纠纷解决机制，推动信访工作重心下移、关口前移。做好国家卫生城市复审迎检工作。

    创新社会治理方式。完善社会服务功能，加大社区投入，加强社区工作者队伍建设。培育各类社会组织，提升承接公共服务和社会公共事务能力。健全基层治理体系，推进城乡社区协商制度建设。深入实施“七五”普法规划。做好法律援助、社区矫正等工作。加强流动人口和特殊人群服务管理。促进民族团结、宗教和睦。支持工会、共青团、妇联等群团改革发展。深化双拥共建，强化国防动员、国防教育和人民防空建设。

    推进“平安马鞍山”建设。推动安全生产领域改革发展，实施安全保障监测体系建设行动，有效遏制较大以上安全生产事故发生。加强食品药品安全监管，创建国家食品安全示范城市。落实粮食安全责任制。健全综合防灾减灾体系，实施地质灾害防御行动。加强应急管理和隐患排查。实施“雪亮工程”，健全立体化、信息化社会治安防控体系。

    各位代表！新时代的号角已经吹响、蓝图已经绘就。摆在各级政府面前的首要任务就是抓落实，一步一个脚印，踏踏实实干好工作，以真抓的实劲、敢抓的狠劲、善抓的巧劲、常抓的韧劲，努力打造人民满意的实干型政府。要强化政治抓落实。深入学习领会习近平新时代中国特色社会主义思想特色社会主义思想，把科学的理论转化为强大的思想武器和行动指南，坚决维护党中央权威和集中统一领导，始终在思想上政治上行动上与以习近平同志为核心的党中央保持高度一致，不折不扣地坚决贯彻党中央、国务院、省委、省政府以及市委重大决策部署。要强化法治抓落实。坚持将政府工作纳入法治化轨道，将权力关进制度的笼子。自觉接受党内监督、人大监督和政协民主监督，广泛听取民主党派、工商联等各界意见，不断提高政府决策科学化、民主化、法治化水平。要强化责任抓落实。以认真负责的工作态度、争创一流的工作业绩、勤勉务实的工作作风，把该担的责任担起来，该抓的工作抓到位，做好政府每一项工作，抓好政府每一项建设，确保政府每一项任务落到实处。要强化效能抓落实。大力弘扬时不我待、只争朝夕的精神，跟上新时代，抢抓新机遇，对全年工作任务进行细化、硬化、量化，制定时间表、路线图，以旬保月、以月保季、以季保年，强化调度、考核、监督和问责，抓紧每一天，干好每件事。要强化服务抓落实。优质的服务是吸引力、凝聚力、竞争力，我们必须强化服务意识，营造“四最”发展环境，构建亲清新型政商关系，吸纳八方客商，激发创业活力，在“零障碍”上求突破，在低成本上下功夫，在高效率上出实招。要强化本领抓落实。既要政治过硬、又要本领高强，全面增强学习本领、政治领导本领、改革创新本领、科学发展本领、依法执政本领、群众工作本领、狠抓落实本领、驾驭风险本领。认真开展以“查问题、出硬招、改彻底”为重点的作风建设大排查，切实改进政府工作作风。

    各位代表！幸福都是奋斗出来的。未来马鞍山的发展，使命艰巨而光荣。让我们更加紧密地团结在以习近平同志为核心的党中央周围，在省委、省政府和市委的坚强领导下，务实苦干、开拓进取，为全力推动“三个走在前列”，全面开创新时代马鞍山现代化建设新局面而努力奋斗！
